# Saturated Fat Restriction for Cardiovascular Disease Prevention: A Systematic Review and Meta-analysis of Randomized Controlled Trials

**DOI:** 10.31662/jmaj.2024-0324

**Published:** 2025-03-21

**Authors:** Satoru Yamada, Tomomi Shirai, Sakiko Inaba, Gaku Inoue, Minami Torigoe, Naoto Fukuyama

**Affiliations:** 1 Diabetes Center, Kitasato University, Kitasato Institute Hospital, Tokyo, Japan; 2 Department of Nutritional Science, Tokyo University of Agriculture, Tokyo, Japan

**Keywords:** saturated fatty acid, cardiovascular mortality, all-cause mortality, systematic review, meta-analysis

## Abstract

**Background:**

The recommendation to limit dietary saturated fat intake is primarily drawn from observational studies rather than randomized controlled trials of cardiovascular disease prevention. Thus, we aimed to investigate the efficacy of saturated fat reduction in preventing mortality and cardiovascular diseases.

**Methods:**

In this systematic review and meta-analysis of randomized controlled trials, Cochrane CENTRAL, PubMed, and Ichu-shi databases were searched for articles up to April 2023. Randomized controlled trials on saturated fat reduction to prevent cardiovascular diseases were selected. Cardiovascular and all-cause mortality and cardiovascular outcomes were evaluated. Changes in electrocardiography or coronary angiography findings were excluded because they could be evaluated arbitrarily. Two or more reviewers independently extracted and assessed the data. A random-effects meta-analysis was performed.

**Results:**

Nine eligible trials with 13,532 participants were identified (2 were primary and 7 were secondary prevention studies). No significant differences in cardiovascular mortality (relative risk [RR] = 0.94, 95% confidence interval [CI]: 0.75-1.19), all-cause mortality (RR = 1.01, 95% CI: 0.89-1.14), myocardial infarction (RR = 0.85, 95% CI: 0.71-1.02), and coronary artery events (RR = 0.85, 95% CI: 0.65-1.11) were observed between the intervention and control groups. However, owing to limited reported cases, the impact of stroke could not be evaluated.

**Conclusions:**

The findings indicate that a reduction in saturated fats cannot be recommended at present to prevent cardiovascular diseases and mortality. Clinical trials are needed to evaluate the effects of saturated fat reduction under the best possible medical care, including statin administration.

**Systematic review registration number:**

This systematic review and meta-analysis was registered with the International Prospective Register of Systematic Reviews (CRD42023428498).

## Background

After the 1977 Senate report, the USA Dietary Reference Intakes recommended restricting saturated fatty acid (SFA) intake ^(1)^ owing to a positive correlation between SFAs and cardiovascular mortality reported in the Seven Countries Study ^(2)^. The UK Public Health Dietary Advice adopted this recommendation in 1983 ^(3)^. SFA intake is associated with serum cholesterol levels. A recent World Health Organization (WHO) report by Mensink stated that replacing SFA with monounsaturated fatty acids (MUFA), polyunsaturated fatty acids (PUFA), and carbohydrates reduces serum total cholesterol and low-density lipoprotein cholesterol (LDL-C) levels ^(4)^. Therefore, SFA restriction may prevent cardiovascular diseases (CVDs). On the basis of 2 observational studies ^(5), (6)^, the American College of Cardiology/American Heart Association (AHA) guideline ^(7)^ recommended SFA restriction. One study showed a positive correlation between SFA intake and all-cause mortality ^(5)^, but the other did not reveal such an association ^(6)^. The Prospective Urban Rural Epidemiology study showed a negative correlation between SFA intake and all-cause mortality ^(6)^. Furthermore, phase 3 trials of cholesteryl ester transfer protein inhibitors have shown that reducing LDL-C does not necessarily prevent CVD. In contrast, in the Investigation of Lipid Level Management to Understand its Impact in Atherosclerotic Events (ILLUMINATE) trial, the administration of torcetrapib significantly decreased LDL-C levels by 24.9% and significantly increased CVD and all-cause mortality ^(8)^. Thus, although many previous randomized controlled trials (RCTs) have proved that “lowering LDL cholesterol is good,” it is essential to determine “how and in whom” this benefit is achieved ^(9)^. According to the lipoprotein subfraction analysis by Bergeron et al. ^(10)^, restriction of SFA intake reduced the large buoyant LDL-C level but not the small dense LDL-C level, which are the most atherogenic lipoproteins. Debate studies on SFA restriction have recently been published in the *Journal of the American College of Cardiology* and the *American Journal of Clinical Nutrition* ^(11), (12), (13), (14)^. In these articles, Krauss and Kris-Etherton disagreed on several points as follows: (1) does reducing SFA intake reduce the incidence of CVD? (2) to what extent is the reduction in LDL-C from lower SFA intake predictive of reduced CVD risk? (3) do dietary SFAs substantially affect factors other than LDL-C that affect CVD risk? and (4) is there a clear rationale for setting a target for maximally reducing dietary SFA? ^(12), (13), (14)^ Among these, the primary concern is, “Does lowering SFA intake reduce the incidence of CVD?” ^(14)^ To address this critical question, we conducted a systematic review and meta-analysis of RCTs to clarify whether SFA restriction prevents CVD and all-cause mortality.

## Methods

### Protocol registration

This systematic review and meta-analysis used a predefined protocol and was registered with the International Prospective Register of Systematic Reviews (PROSPERO) (CRD42023428498) in accordance with the Preferred Reporting Items for Systematic Reviews and Meta-Analyses guidelines. This study was approved by the Kitasato Institute Hospital Research Ethics Committee (number 22057). Our research question was whether SFA restriction could prevent CVDs. The outcomes of previous studies included soft end points that were questionable, such as chest pain without electrocardiographic changes ^(15)^. Hence, we selected cardiovascular mortality as the primary outcome. Moreover, we planned to include all-cause mortality, myocardial infarction (MI), any coronary artery events, stroke, and a composite of non-fatal MI, non-fatal stroke, and cardiovascular death as the secondary outcomes.

### Data sources and searches

We searched the Cochrane CENTRAL, PubMed, and Ichu-shi databases from inception (Cochrane CENTRAL 1996, PubMed 1966, and Ichu-shi 1964) to April 2023. We selected these 3 databases according to guidelines for systematic reviews of the Medical Information Network Distribution Service Center, Japan Council for Quality Health Care (Minds Centre) which is an official member of the Guidelines International Network ^(16)^.

### Study selection

We selected only RCTs in the literature review because of the inherent biases in observational research. We did not set any limitation regarding language. Relevant articles were identified according to the literature search strategies developed by the collaborators (librarians) (Supplementary Data S1, S2, and S3). Furthermore, we searched the reference lists of previous studies. The following inclusion criteria were implemented to identify studies for inclusion in our systematic review and meta-analysis: (1) RCT design, (2) SFA restriction as the intervention, (3) CVD as the outcome, and (4) adults as the target population. Two investigators (SY and TS) independently screened the titles and abstracts of articles according to the inclusion criteria. In the next stage of full-text screening, the authors (SY and TS) reached a consensus on which studies should be included or excluded. Disagreements regarding the results of the screened studies were resolved by discussion with a third author (NF).

We used Rayyan software (Qatar Computing Research Institute) for the screenings. The following information from the eligible studies was extracted by 2 authors (SY and NF): authors, publication year, country, sample characteristics, sample size, length of follow-up, cardiovascular outcomes, SFA energy percentage (if available), and lipid profile (if available).

### Quality assessment

We used the updated Cochrane Collaboration’s risk of bias tool to assess the risk of bias (RoB2) ^(17)^. This tool evaluates 5 possible sources of bias leading to determine the overall risk: bias arising from the randomization process, bias due to deviation from intended interventions, bias due to missing outcome data, bias in measurement of the outcome, and bias in selection of the reported result.

### Statistical analysis

For the meta-analysis, each odds ratio (OR) was combined with SFA restriction, and the pooled ORs with 95% confidence interval (CI) were calculated using the random-effects model. Heterogeneity among the studies was evaluated using I^2^ statistics. Funnel plots were used to estimate publication bias. All statistical analyses were performed using R (The R Foundation for Statistical Computing, Vienna, Austria).

## Results

### Study selection

**Figure 1.**
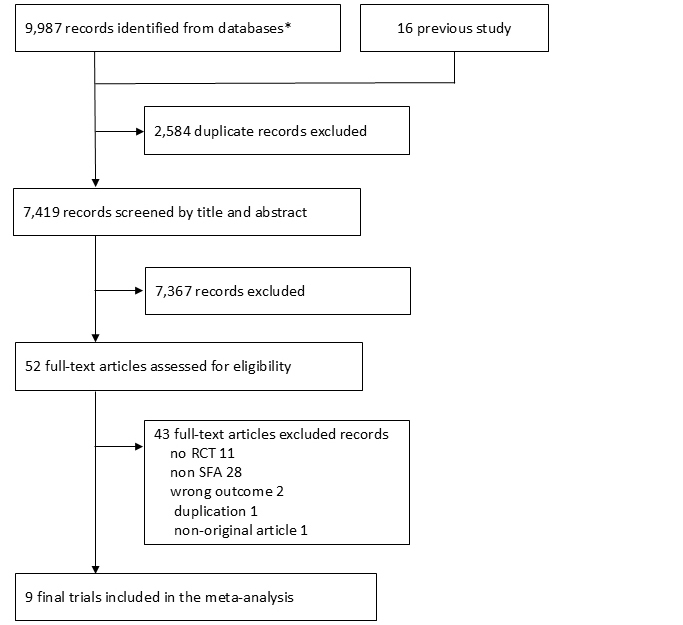
Results of the systematic search and study selection process. *Indicates the PubMed, Cochrane CENTRAL, and Ichusi databases. A total of 9987 studies were included: 3969 from PubMed, 5919 from Cochrane CENTRAL, and 99 from Ichusi.

A total of 7,419 articles were identified during our search; 52 were assessed for their eligibility for inclusion (Figure 1). Finally, 9 studies were included in the systematic review and meta-analysis ^(15), (18), (19), (20), (21), (22), (23), (24), (25)^.

### Characteristics of the included studies

Table 1 lists the characteristics of the included studies. The sample sizes in these studies ranged from 50 to 9,057. Five studies were conducted in European countries ^(15), (18), (19), (21), (23)^, 2 in the USA ^(20), (22)^, 1 in Australia ^(24)^, and 1 in India ^(25)^. Only 2 studies were primary prevention trials from the USA ^(20), (22)^; the remaining 7 were secondary prevention trials. Thus, we did not perform a sub-analysis of primary prevention. Only 3 studies were on stroke ^(20), (23), (25)^, and the incidence of stroke was almost zero in 2 of those studies ^(23), (25)^. Therefore, we did not perform a meta-analysis of stroke and a composite of non-fatal MI, non-fatal stroke, and cardiovascular death. Only 1 study included participants who received statins, and it showed no difference in CVD events between the intervention and control groups ^(25)^. In the other 8 studies, none of the participants used statins. Thus, we did not perform a sub-analysis of statin use. Only 2 studies included female participants, showing no differences in cardiovascular outcomes between the intervention and control groups ^(22), (25)^. We did not conduct a sub-analysis of sex-based differences.

**Table 1.** Characteristics of the Included Studies.

First author (publication year)	Study name	Country	Population	Number of participants (intervention/control)	Sex	Statin user	Diet in the intervention group	Diet in control group	Difference in SFA intake	Follow-up year	Primary outcome
**Rose et al. ^(18)^ (1965)**	St. Mary Hosp	UK	Post-MI or angina	54/26	n.a.	None	Avoid animal fat with supplementation of olive or corn oil	n.a.	n.a.	2	Sudden death or MI
**Leren ^(19)^ (1966)**	Oslo Diet Heart	Norway	Post-MI	206/206	Male 100%	None	SFA8.5%	n.a.	n.a.	5	CHD relapse
**Research Committee ^(15)^ (1968)**	MRC	UK	Post-MI	199/194	Male 100%	None	85g soya oil SFA15.3%	No soya oil SFA 39.4%	24.1% energy	5	MI
**Dayton et al. ^(20)^ (1969)**	Los Angeles Veterans Administration	USA	Veterans domicile	424/422	Male 100%	None	Linoleic acid 38.4% of fat	Linoleic acid 10.0% of fat	n.a.	8	Sudden death or definite MI
**Burr et al. ^(21)^ (1989)**	DART	UK	Post-MI	1018/1015	Male 100%	None	P:S=0.78 SFA18.1%	P:S=0.44 SFA24.3%	6.2% energy	2	MI
**Frantz et al. ^(22)^ (1989)**	Minnesota Coronary Survey	USA	Mental hospital or nursing home	4541/4516	Female 41.5%, male 48.5%	None	SFA9%	SFA18%	9.1% energy	4.5	MI, sudden death, or death
**Watts et al. ^(23)^ (1992)**	STARS	UK	CAG	26/24	Male 100%	None	SFA9%	SFA16%	7.0% energy	3.25	CAG change
**Ramsden et al. ^(24)^ (2013)**	Sydney Diet Heart Study	Australia	Post-ACS	221/237	Male 100%	None	PUFA15.4%, SFA9.3%	PUFA8.4%, SFA13.5%	4.2% energy	5	Death
**Vijayakumar et al. ^(25)^ (2016)**	Amrita Institute	India	Stable CAD	100/100	Female 6.6%, male 93.4%	all	Sunflower oil	Coconut oil	n.a.	2	Anthropometric change, lipid profile

ACS: acute coronary syndrome; CAD: coronary artery diseases; CAG: coronary angiography; CHD: coronary heart disease; MI: myocardial infarction; P:S: polyunsaturated fatty acid:saturated fatty acid; PUFA: polyunsaturated fatty acid; SFA: saturated fatty acid.

### Results of the meta-analysis

**Figure 2.**
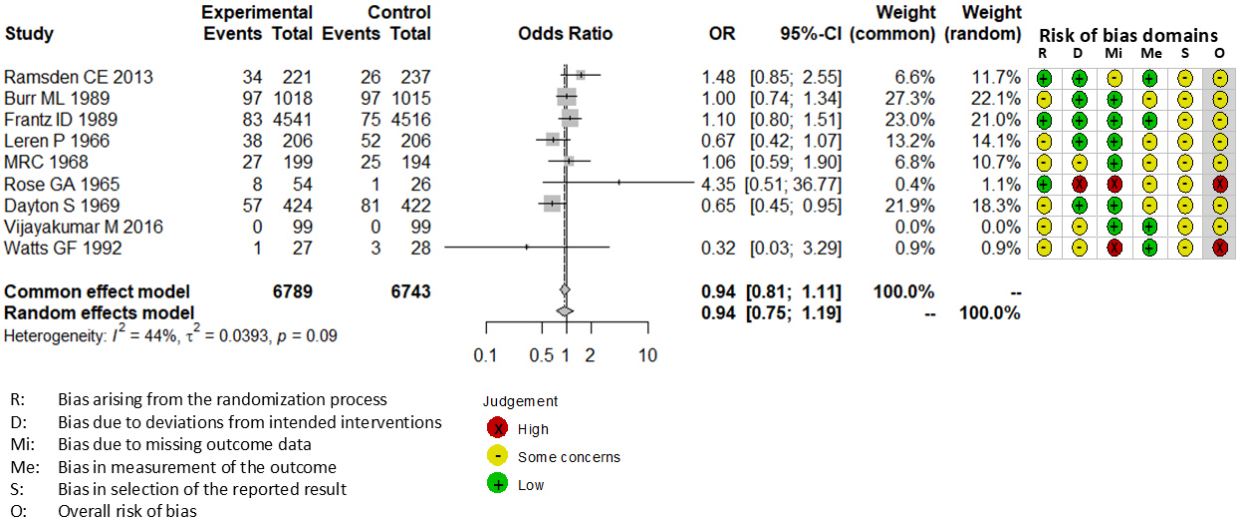
Forest plots of saturated fatty acid reduction trials on cardiovascular mortality. No statistically significant reduction was noted. CI: confidence interval; OR: odds ratio.

**Figure 3.**
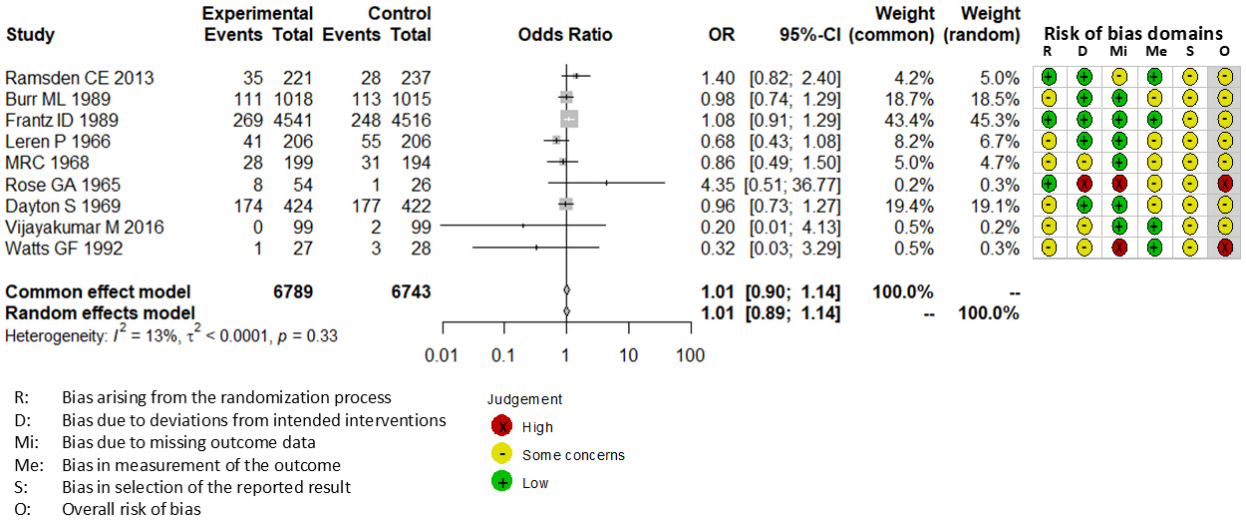
Forest plots of saturated fatty acid reduction trials on all-cause mortality. No statistically significant reduction was noted. CI: confidence interval; OR: odds ratio.

**Figure 4.**
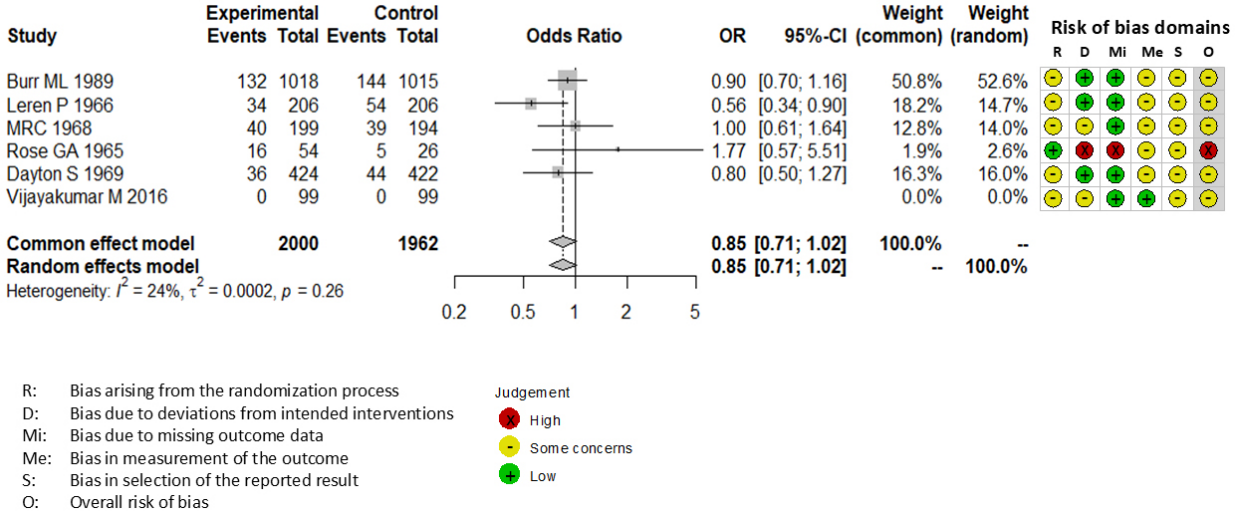
Forest plots of saturated fatty acid reduction trials on myocardial infarction. No statistically significant reduction was noted. CI: confidence interval; OR: odds ratio.

**Figure 5.**
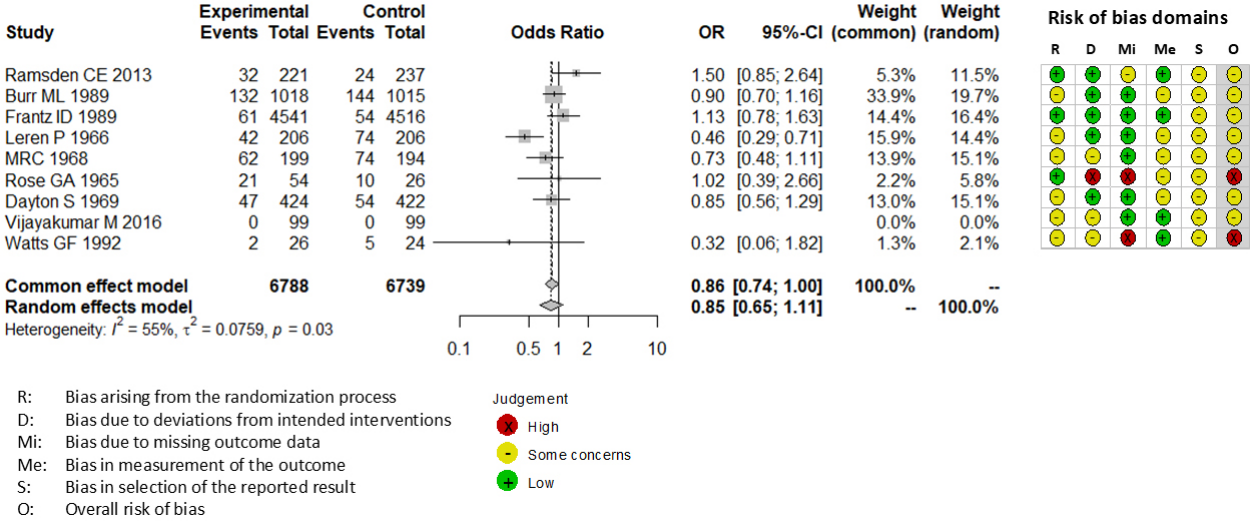
Forest plots of saturated fatty acid reduction trials on any coronary artery event. No statistically significant reduction was noted. CI: confidence interval; OR: odds ratio.

**Figure 6.**
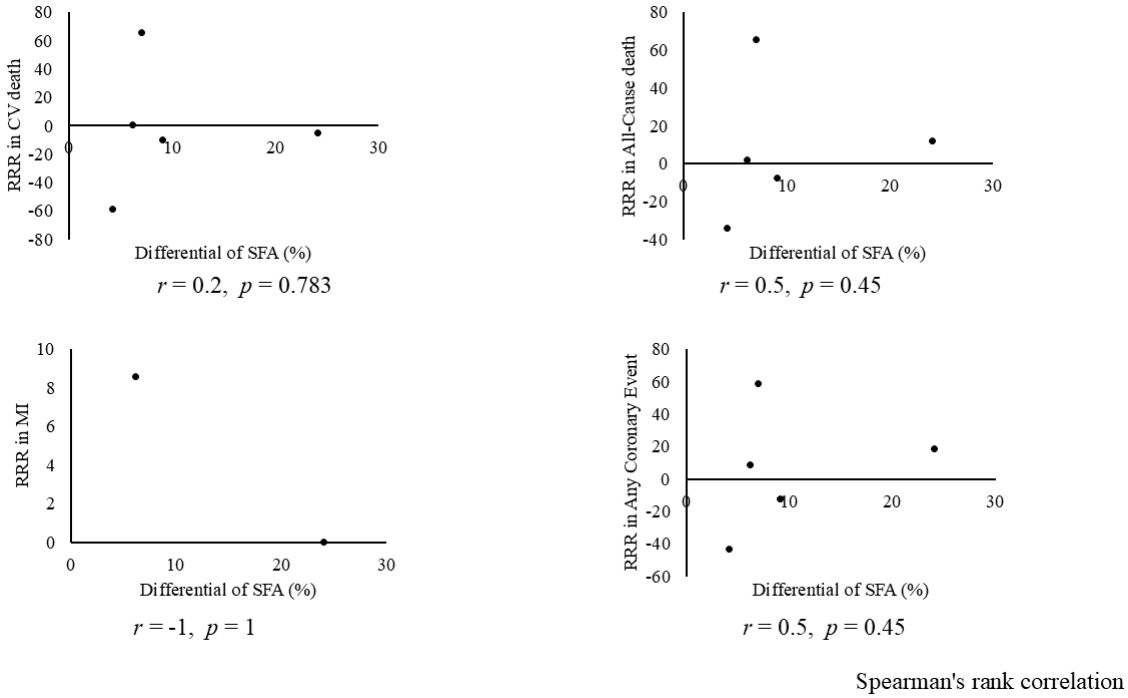
Relationship between the reduction in SFA intake and RRR of each outcome. No association suggesting dose dependency was observed between the differential of SFA (x axis) (%) and RRR of each outcome (y axis). CVD: cardiovascular disease; MI: myocardial infarction; RRR: relative risk reduction; SFA: saturated fatty acid.

**Figure 7.**
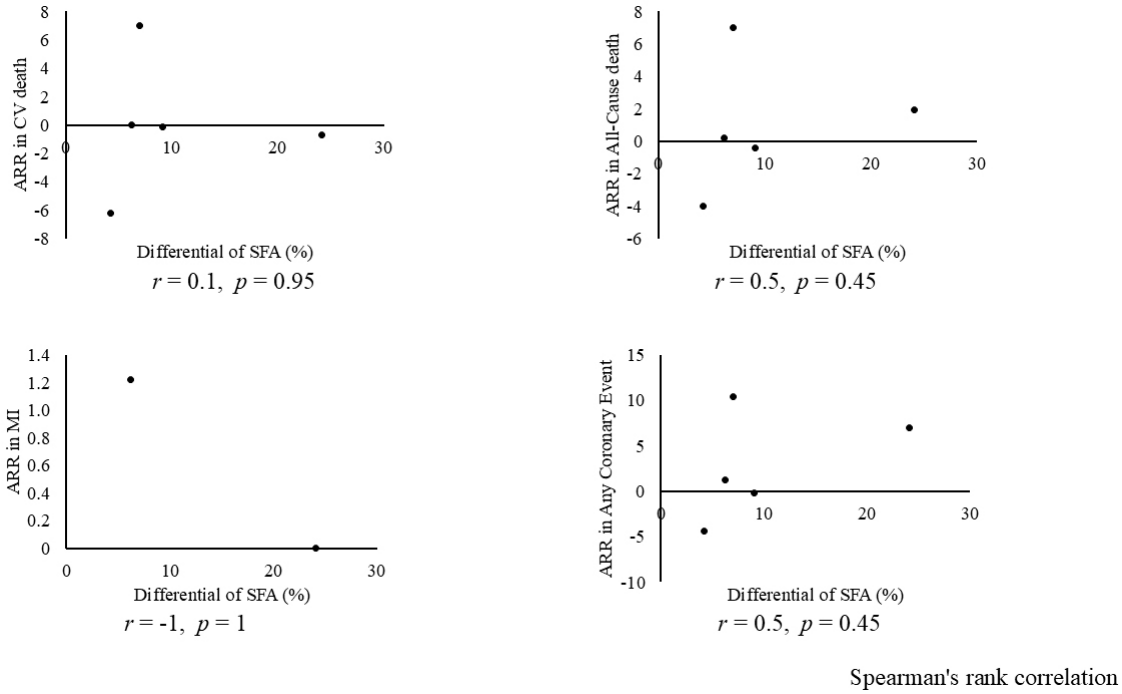
Relationship between the reduction in saturated fatty acid intake and ARR of each outcome. No association suggesting dose dependency was observed between the differential of SFA (x axis) (%) and ARR of each outcome (y axis). ARR: absolute risk reduction; CVD: cardiovascular disease; MI: myocardial infarction.

**Figure 8.**
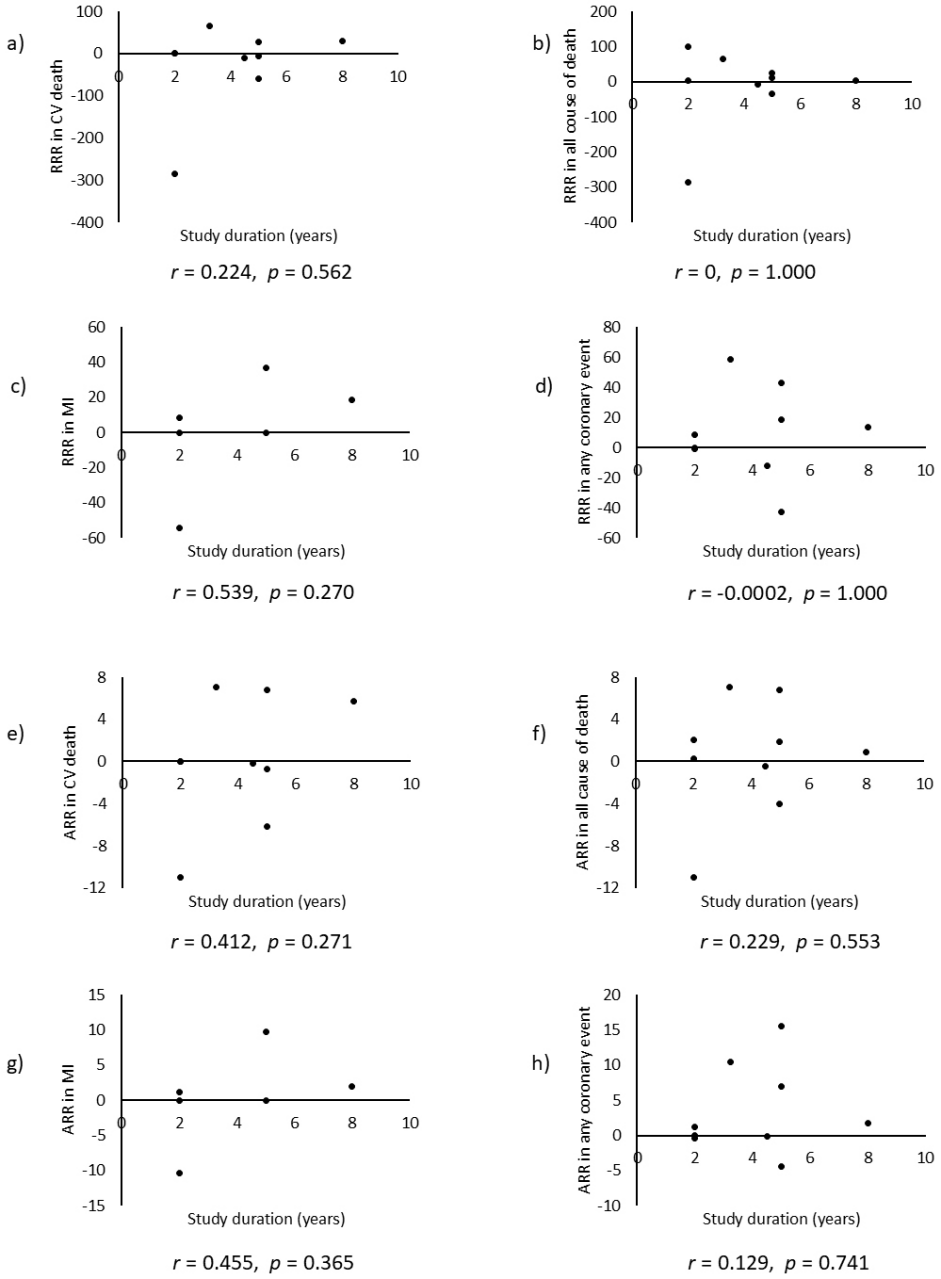
Relationship between study duration (x axis) and RRR or ARR of each outcome (y axis). According to Pearson’s product-moment correlation analysis, no association between study duration and RRR [upper 4 panels: a)-d)] or ARR [lower 4 panels: e)-h)] was observed. ARR: absolute risk reduction; CVD: cardiovascular disease; MI: myocardial infarction; RRR: relative risk reduction.

Combining all trials, the pooled risk reduction for cardiovascular mortality was not significant (OR = 0.94, 95% CI: 0.75-1.19) (Figure 2). In addition, no reduction in all-cause mortality was noted (OR = 1.01, 95% CI: 0.89-1.14) (Figure 3). Furthermore, no statistically significant reductions in OR were observed regarding MI (OR = 0.85, 95% CI: 0.71-1.02) or any coronary artery events (OR = 0.85, 95% CI: 0.65-1.11) (Figure 4 and 5). Given the possibility that some studies had insufficient SFA restrictions for statistical significance, we plotted the relationship between the difference in SFA intake between the 2 groups and the relative risk reduction (RRR) or absolute risk reduction (ARR) for each trial. No association was observed between the difference in SFA intake and RRR or ARR for any outcomes (Figure 6 and 7). Furthermore, no association was observed between the study duration and RRR or ARR for any outcome, considering metabolic memory and legacy effect (Figure 8).

### Test of bias

The RoB2 tool revealed that none of 9 trials included in our meta-analysis exhibited a low risk of bias. Seven trials exhibited some concerns ^(15), (19), (20), (21), (22), (24), (25)^, and 2 trials exhibited a high risk of bias ^(18), (23)^. All trials were not pre-registered in a clinical trial registry such as Clinical Trials Gov and UMIN.

**Figure 9.**
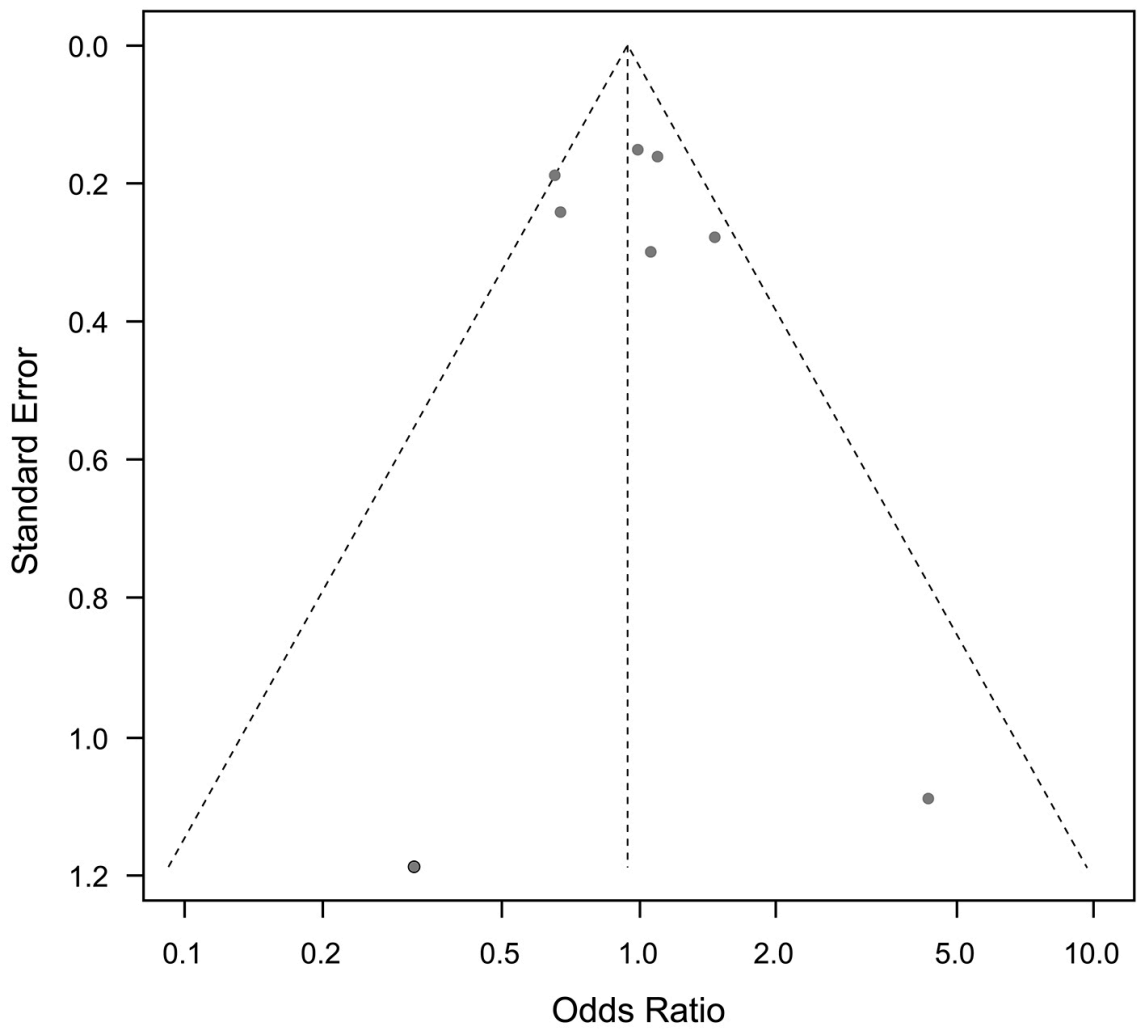
Funnel plots of saturated fatty acid reduction trials on cardiovascular mortality. No publication bias was noted.

**Figure 10.**
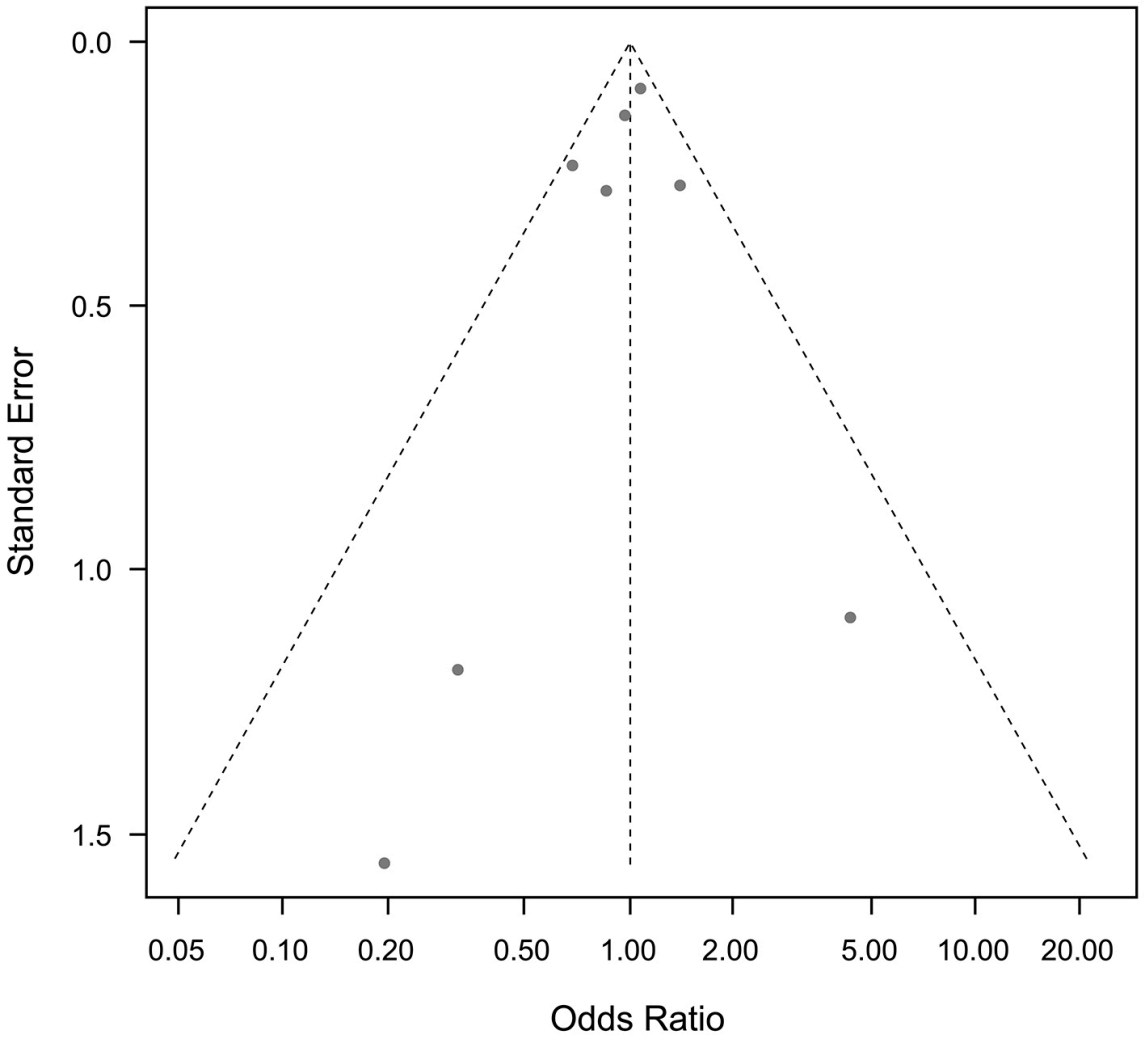
Funnel plots of saturated fatty acid reduction trials on all-cause mortality. No publication bias was noted.

Funnel plots for cardiovascular mortality and all-cause mortality, and any other outcomes, showed no publication bias (Figure 9 and 10).

## Discussion

In the present meta-analysis, we found no statistically significant evidence supporting CVD prevention through SFA reduction. Several previous meta-analysis have investigated the role of SFA restriction for CVD prevention ^(26), (27), (28)^, and these have been cited as evidence supporting the recommendation of SFA restriction by the European Society of Cardiology/European Atherosclerosis Society ^(29)^. Compared with those of previous meta-analyses ^(26), (27), (28)^, the primary strengths of our study lie in its rigorous inclusion criteria and the specification of its literature search strategy.

### Previous meta-analysis 1

Mozaffarian et al. ^(26)^ reported a significant reduction in CVD events with SFA restriction. The discrepancy between the present meta-analysis and previous studies is due to differences in the included studies (Table 2). Given Mozaffarian et al. ^(26)^ did not describe their strategy for literature search, we could not follow their search and study selection strategy. However, the Finnish Mental Hospital Studies ^(30), (31)^, which were included in the analysis by Mozaffarian et al. ^(26)^, were excluded from the present study because they were non-randomized cross-over trials. Moreover, in these studies ^(30), (31)^, the study population comprised patients admitted to 2 mental hospitals, and patients could be discharged or admitted during the study period. In a study involving 676 hospitalized male patients, only 36.4% (n = 246) remained hospitalized during both interventional and control periods ^(30)^. In a study involving female subjects, only 20.6% (122 of 591) remained hospitalized during both periods ^(31)^. Most of these studies were non-randomized and did not follow a cross-over design ^(30), (31)^. In addition, differences in patient characteristics and treatment policies other than diet, and in the interventional and conventional diets between the 2 mental hospitals, may have influenced the results ^(30), (31)^. Thus, we believe that these studies should be excluded from our analysis. Conversely, several studies that were included in our meta-analysis were not included in the study by Mozaffarian et al. ^(26)^ For example, the study by Rose et al. ^(18)^ and the Sydney Diet Heart Study ^(24)^ were among the top 2 negative studies (Figure 2) that would have provided valuable contributions to the analysis by Mozaffarian et al. ^(26)^

**Table 2.** Characteristics of the Excluded Studies That Were Included in Other Meta-Analyses.

First author (publication year)	Study name	Population	Reason of exclusion
**Turpeinen et al.^(30)^ (1979)**	Finnish Mental Hospital Study (Men)	Patients in mental hospital	Non-randomized study
			Cross-over design without evaluation of carry-over effect
			Most subjects (63.6%) participated in single arm only (not crossed-over)
**Miettinen et al.^(31)^ (1983)**	Finnish Mental Hospital Study (Women)	Patients in mental hospital	Non-randomized study
			Cross-over design without evaluation of carry-over effect
			Most subjects (79.4%) participated in single arm only (not crossed-over)
**Black et al.^(32)^ (1994)**	n.a.	Previous skin cancer	Total fat reduction intervention but not SFA
			No data for CVD or death
**Houtsmuller et al.^(33), (34), (35)^ (1980)**	n.a.	Diabetes	Unknown definition of cardiac ischemia
			Glucose improvement only in women
**Ley et al.^(36)^ (2004)**	n.a.	Prediabetes	Total fat reduction intervention but not SFA
			No data for CVD or death
**Moy et al.^(37)^ (2001)**	n.a.	Healthy siblings with a family history of CHD	No data for CVD or death
**Howard et al.^(38)^ (2006)**	Women’s Health Initiative	Postmenopausal women	Total fat reduction intervention but not SFA

CVD: cardiovascular diseases; n.a: not applicable; SFA: saturated fatty acid.

### Previous meta-analysis 2

In the presidential advisory of the AHA by Sacks et al. ^(27)^, the literature search strategy was not described, similarly to the study by Mozaffarian et al. ^(26)^ Moreover, those authors included the Finnish Mental Hospital Studies ^(30), (31)^, which are non-randomized cross-over trials. In addition to being a non-randomized trial, we believe that cross-over trials are inappropriate for cardiovascular outcome studies owing to potential carry-over effects, referred to as a metabolic memory or legacy effect (Table 2).

### Previous meta-analysis 3

Regarding the Cochrane Systematic Review by Hooper et al. ^(28)^, the results of cardiovascular (relative risk [RR] = 0.96; 95% CI: 0.90-1.03) and all-cause mortalities (RR = 0.94; 95% CI: 0.78-1.13) are similar to those of our present study (OR = 0.94; 95% CI: 0.75-1.19 for cardiovascular mortality and OR = 1.01; 95% CI: 0.89-1.14 for all-cause mortality). However, Hooper et al. reported a statistically significant reduction (p = 0.03) in combined cardiovascular events with SFA reduction (RR = 0.83, 95% CI: 0.70-0.98) ^(28)^, which differs from our findings. Although the authors described the literature search strategy, they included several studies that were not eligible for inclusion in our analysis (Table 2) ^(32), (33), (34), (35), (36), (37), (38)^. First, Black et al. ^(32)^ reported actinic keratosis as the primary outcome in patients with skin cancer, rather than CVD. Furthermore, dietary intervention focused on a reduction in total fat but not in SFA. Second, the primary outcome in the study by Houtsmuller et al. ^(33)^ was diabetic microangiopathy or retinopathy, not CVD. Although other reports in the analysis by Houtsmuller et al.^(34), (35)^ have reported cardiovascular outcomes, the definition or criteria for cardiac ischemia were not provided. Furthermore, a statistically significant difference in glucose levels was observed between the 2 groups only among female patients ^(34)^. However, this phenomenon could not be explained in terms of causality. Therefore, we deemed this study to be too unique for inclusion in our analysis. Third, the study by Ley et al. ^(36)^ did not report the incidence of cardiovascular events as an outcome. The outcome of this study was CVD risk factors rather than CVD events. Moreover, the dietary intervention in this study focused on a reduction in total fat but not in SFA ^(36)^. Fourth, Moy et al. ^(37)^ did not report the incidence of cardiovascular events. The outcome of their study was adherence to dietary counseling, rather than CVD events ^(37)^. Finally, the intervention of the Women’s Health Initiative focused on a reduction in total fat but not in SFA ^(38)^. In this largest RCT on total fat reduction, no difference in cardiovascular outcomes was observed among the whole study participants. However, deterioration in cardiovascular outcomes and all-cause mortality was reported in secondary prevention subpopulations despite an approximately 4 mg/dL reduction in LDL-C levels ^(38), (39)^. To summarize, “lowering LDL cholesterol is good,” but determining “how and in whom” is crucial ^(9)^.

### Narrow evidence of SFA restriction

Owing to the limitations of previous meta-analyses on SFA reduction, we conducted a renewed meta-analysis in a methodologically understandable manner. Although we did not observe statistically significant CVD prevention due to SFA reduction, several studies included in our meta-analysis reported positive results with SFA reduction ^(15), (19), (20), (23)^. In fact, although the results for MI and any coronary artery events (Figure 4 and 5) did not reach statistical significance in our present meta-analysis, our findings suggest a trend toward effectiveness. Therefore, further RCTs are needed before recommendations can be fully supported or dismissed. Furthermore, in previous studies showing positive outcomes ^(15), (19), (20), (23)^, statins were not prescribed to the participants. In the only study in which all participants received statins, no significant difference was observed in CVD events (2.0% of participants experienced non-fatal MI and 0 experienced stroke and cardiovascular death in both groups) or LDL-C level (89.6 ± 28.9 mg/dL in intervention and 91.0 ± 21.8 mg/dL in control groups) ^(25)^. Regarding statin administration, there is no evidence to support that SFA reduction can further improve LDL-C levels or prevent CVD. Future studies should evaluate the effectiveness of SFA restriction under the best possible medical care including statin administration. Moreover, only 2 studies that included female participants showed no differences in cardiovascular outcomes between the intervention and control groups ^(22), (25)^. Thus, there is no evidence to support the efficacy of SFA reduction in women. Future studies should evaluate the effectiveness of SFA restriction in both sexes. According to the WHO report by Mensink, the replacement of 1% energy from SFA with PUFA reduced the LDL-C level by 0.055 mmol/L (2.1 mg/dL), whereas MUFA and carbohydrates reduced the LDL-C level by 0.042 mmol/L (1.6 mg/dL) and 0.033 mmol/L (1.3 mg/dL), respectively ^(4)^. Such small changes in LDL-C levels do not seem to affect cardiovascular outcomes and serum lipid profiles with statin administration, which can reduce the LDL-C level by approximately 50 mg/dL ^(40)^. Furthermore, if the LDL-C level remains above the target level with statin administration, ezetimibe and PCSK9 inhibitors can be administered ^(41)^.

### Limitations

Our study has some limitations. First, RCTs on dietary interventions cannot be completely blinded, which introduces a proportional risk of bias. However, this is a common limitation in studies on dietary interventions. Second, all studies were not pre-registered in a clinical trial registry. Thus, we could not examine the consistency between the prespecified analysis plan and actual analysis conducted, further increasing the risk of bias. Third, except for 1 study, all other studies did not involve statin administration; therefore, the effects of SFA restriction under the current best possible medical care have been scarcely investigated. Fourth, except for 2 studies, all other studies did not involve female participants; therefore, the effects of SFA restriction in women have been scarcely investigated. Fifth, most studies were conducted in Western countries and failed to examine racial or ethnic differences. Finally, many studies have been conducted on interventions that replaced animal fats with vegetable fats and did not analyze the impact of individual SFAs with different chain lengths. For example, the difference between milk and meat fats remains unclear.

## Conclusions

The study findings indicate that the evidence available from RCTs does not support SFA restriction for the prevention of CVDs. Specifically, regarding statin administration, only 1 RCT showed no significant difference in CVD events and LDL-C levels between the intervention and control groups. To maintain the recommendation for SFA reduction in CVD prevention and LDL-C level improvement, further clinical trials are needed to evaluate its effects alongside statin administration. In such trials, researchers should address the sex-related differences, which would address the knowledge gaps that have contributed to the controversies.

## Article Information

### Conflicts of Interest

Satoru Yamada received honoraria for lectures from Elly Lilly and Eat Fun Health Association and has stock in LOCABO Inc. Tomomi Shirai, Sakiko Inaba, Gaku Inoue, Minami Torigoe, and Naoto Fukuyama declare no conflicts of interest.

### Sources of Funding

This study was funded by a Kitasato Institute Hospital Research Grant (Grant Number 8125).

### Acknowledgement

We thank Ms. Kyoko Abe on behalf of the librarian team at Kitasato University Shirokane Library for advice regarding the literature search strategy. We also thank Editage (www.editage.jp) for the English language editing. This study was registered with PROSPERO (CRD42023428498). The collaborators (librarian team) were Kyoko Abe, Riko Tajima, Teppei Hashimoto, Seiko Yano, and Eiko Hirose. We used Rayyan software (Qatar Computing Research Institute) for the screenings.

### Author Contributions

Satoru Yamada and Tomomi Shirai contributed equally to this work. Satoru Yamada, Tomomi Shirai, Sakiko Inaba, Minami Torigoe, and Naoto Fukuyama were responsible for the conceptualization. Satoru Yamada and Tomomi Shirai were responsible for the methods. Satoru Yamada, Tomomi Shirai, Sakiko Inaba, and Minami Torigoe undertook the literature search. Satoru Yamada, Tomomi Shirai, and Naoto Fukuyama performed the data screening. Satoru Yamada, Tomomi Shirai, Sakiko Inaba, and Gaku Inoue undertook the formal analysis. Satoru Yamada undertook the writing―original draft preparation and guaranteed the work. Naoto Fukuyama supervised the work. The guarantor accepts full responsibility for the work and/or the conduct of the study, had access to the data, and controlled the decision to publish. The corresponding author attests that all listed authors meet the authorship criteria and that no others meeting the criteria have been omitted. All authors, external and internal, had full access to all the data (including statistical reports and tables) in the study and take responsibility for the integrity and accuracy of the data. All authors read and approved the final manuscript. SY and TS equally contributed to this work.

### Approval by Institutional Review Board (IRB)

The protocol for this study has been approved by the Kitasato Institute Hospital Research Ethics Committee (Approval Number 22057).

### Informed Consent

The ethics committee waived the need for informed consent because this is a systematic review.

### Data Availability

The datasets generated and/or analyzed during the present study are available from the corresponding author on reasonable request.

## Supplement

Supplementary Materials
