# Supplementary material for: Saturated Fat Restriction for Cardiovascular Disease Prevention: A Systematic Review and Meta-analysis of Randomized Controlled Trials
Source: JMA J. 2025 Mar 21;8(2):395–407. doi: 10.31662/jmaj.2024-0324 (PMC12095860; doi:10.31662/jmaj.2024-0324)
Supplement: Supplementary Materials [file 2433-3298-8-2-0395-s001.pdf]

Saturated Fat Restriction for Cardiovascular Disease Prevention: Systematic Review and Meta-Analysis of Randomised Controlled Trials

Supplementary Materials

Supplementary Data S1. Literature Search Strategy for PubMed

#1 (("dyslipidemias"[MeSH Terms] OR "dyslipid*"[Title/Abstract] OR "dyslipoproteinemia*"[Title/Abstract] OR "hyperlipemia*"[Title/Abstract] OR "hyperlipid*"[Title/Abstract] OR "lipemia*"[Title/Abstract] OR "lipidemia*"[Title/Abstract] OR "cholesteremia"[Title/Abstract] OR "cholesterinemia"[Title/Abstract] OR "elevated cholesterol*"[Title/Abstract] OR "high cholesterol level*"[Title/Abstract] OR "hyper beta lipoproteinemia"[Title/Abstract] OR "hypercholesteremia*"[Title/Abstract] OR "hypercholesterol*"[Title/Abstract] OR "diabetes mellitus"[MeSH Terms] OR "diabet*"[Title/Abstract] OR "obesity"[MeSH Terms] OR "adiposi*"[Title/Abstract] OR "corpulenc*"[Title/Abstract] OR "fatness"[Title/Abstract] OR "obesity"[Title/Abstract] OR "overweight"[Title/Abstract]) AND ((("fatty acids"[MeSH Terms] OR "aliphatic acid"[Title/Abstract:~0] OR "aliphatic acids"[Title/Abstract:~0] OR "fa"[Title/Abstract] OR "fatty acid"[Title/Abstract:~1] OR "fatty acids"[Title/Abstract:~1] OR "scfa"[Title/Abstract] OR "vfa"[Title/Abstract] OR "acid short chain fatty"[Title/Abstract] OR "animal fat*"[Title/Abstract] OR "ba"[Title/Abstract] OR "butanoic acid*"[Title/Abstract] OR "butyric acid*"[Title/Abstract] OR "acetic acid*"[Title/Abstract] OR "ethanoic acid*"[Title/Abstract] OR "vinegar"[Title/Abstract] OR "triglycerides"[MeSH Terms] OR "tg"[Title/Abstract] OR "triacylglycerol*"[Title/Abstract] OR "triglyceride*"[Title/Abstract]) AND ("nutrition therapy"[MeSH Terms] OR "nutrition*"[Title/Abstract] OR "diet therapy"[MeSH Terms] OR "diet*"[Title/Abstract] OR "food*"[Title/Abstract])) OR ("diet, vegetarian"[MeSH Terms] OR "plant based diet*"[Title/Abstract] OR "plant based nutrition"[Title/Abstract] OR "vegetarian*"[Title/Abstract] OR "vegan*"[Title/Abstract] OR "macrobiotic diet*"[Title/Abstract] OR "diet macrobiotic"[Title/Abstract] OR "fat control diet"[Title/Abstract] OR "dietary fats"[MeSH Terms] OR "dietary fat"[Title/Abstract:~0] OR "dietary fats"[Title/Abstract:~0] OR "dietary oil*"[Title/Abstract])) AND ("mortality"[MeSH

Terms] OR "cause of death"[MeSH Terms] OR "mortalit*"[Title/Abstract] OR "death"[Title/Abstract] OR "fatalit*"[Title/Abstract] OR "lethalit*"[Title/Abstract] OR "stroke"[MeSH Terms] OR "cerebral crisis"[Title/Abstract] OR "cerebral vascular accident*"[Title/Abstract] OR "cerebrovascular accident*"[Title/Abstract] OR "brain vascular accident*"[Title/Abstract] OR "vascular accident brain"[Title/Abstract] OR "stroke*"[Title/Abstract] OR "apoplectic fit"[Title/Abstract] OR "apoplectic ictus"[Title/Abstract] OR "apoplectic seizure"[Title/Abstract] OR "apoplectic cerebral"[Title/Abstract] OR "apoplectic attack*"[Title/Abstract] OR "apoplexy"[Title/Abstract] OR "cva"[Title/Abstract] OR "cvas"[Title/Abstract] OR "ais"[Title/Abstract] OR "anterior cerebral circulation infarction"[Title/Abstract:~0] OR "brain infarct*"[Title/Abstract] OR "infarct brain"[Title/Abstract] OR "infarcts brain"[Title/Abstract] OR "infarction brain"[Title/Abstract] OR "infarcted brain"[Title/Abstract] OR "brain venous infarction*"[Title/Abstract] OR "ci"[Title/Abstract] OR "cerebral infarct*"[Title/Abstract] OR "watershed infarct*"[Title/Abstract] OR "risk"[MeSH Terms] OR "adverse effects"[MeSH Subheading])) OR (("cardiovascular diseases"[MeSH Terms] OR "cardiovascular disease*"[Title/Abstract] OR "cvd"[Title/Abstract] OR "ami"[Title/Abstract] OR "cardiac infarction"[Title/Abstract] OR "cardiovascular stroke*"[Title/Abstract] OR "coronary infarction*"[Title/Abstract] OR "heart attack*"[Title/Abstract] OR "mi"[Title/Abstract] OR "myocardial infarct*"[Title/Abstract] OR "omi"[Title/Abstract] OR "ihd"[Title/Abstract] OR "ischemic cardiomyopathy"[Title/Abstract] OR "ischemic heart disease*"[Title/Abstract] OR "myocardial ischemia*"[Title/Abstract] OR "heart disease ischemic"[Title/Abstract] OR "ischemia myocardial"[Title/Abstract] OR "ap"[Title/Abstract] OR "angina pectoris"[Title/Abstract] OR "angor pectoris"[Title/Abstract] OR "stenocardia*"[Title/Abstract] OR "cad"[Title/Abstract] OR "chd"[Title/Abstract] OR

"coronary artery disease*"[Title/Abstract] OR "coronary disease*"[Title/Abstract] OR "coronary heart disease*"[Title/Abstract] OR "coronary syndrome*"[Title/Abstract] OR "acs"[Title/Abstract] OR "hypertension"[Title/Abstract] OR "high blood pressure*"[Title/Abstract] OR "hypertonia*"[Title/Abstract] OR "ht"[Title/Abstract] OR "blood pressure high"[Title/Abstract] OR "blood clot*"[Title/Abstract] OR "prethrombotic state"[Title/Abstract] OR "thrombose*"[Title/Abstract] OR "thrombosis"[Title/Abstract] OR "thrombus"[Title/Abstract] OR "thromboembolism*"[Title/Abstract] OR "embolism*"[Title/Abstract] OR "embolus"[Title/Abstract] OR "emboli"[Title/Abstract]) AND ((("fatty acids"[MeSH Terms] OR "aliphatic acid"[Title/Abstract:~0] OR "aliphatic acids"[Title/Abstract:~0] OR "fa"[Title/Abstract] OR "fatty acid"[Title/Abstract:~1] OR "fatty acids"[Title/Abstract:~1] OR "scfa"[Title/Abstract] OR "vfa"[Title/Abstract] OR "acid short chain fatty"[Title/Abstract] OR "animal fat*"[Title/Abstract] OR "ba"[Title/Abstract] OR "butanoic acid*"[Title/Abstract] OR "butyric acid*"[Title/Abstract] OR "acetic acid*"[Title/Abstract] OR "ethanoic acid*"[Title/Abstract] OR "vinegar"[Title/Abstract] OR "triglycerides"[MeSH Terms] OR "tg"[Title/Abstract] OR "triacylglycerol*"[Title/Abstract] OR "triglyceride*"[Title/Abstract]) AND ("nutrition therapy"[MeSH Terms] OR "nutrition*"[Title/Abstract] OR "diet therapy"[MeSH Terms] OR "diet*"[Title/Abstract] OR "food*"[Title/Abstract])) OR ("diet, vegetarian"[MeSH Terms] OR "plant based diet*"[Title/Abstract] OR "plant based nutrition"[Title/Abstract] OR "vegetarian*"[Title/Abstract] OR "vegan*"[Title/Abstract] OR "macrobiotic diet*"[Title/Abstract] OR "diet macrobiotic"[Title/Abstract] OR "fat control diet"[Title/Abstract] OR "dietary fats"[MeSH Terms] OR "dietary fat"[Title/Abstract:~0] OR "dietary fats"[Title/Abstract:~0] OR "dietary oil*"[Title/Abstract])))

#2 "Randomized Controlled Trial"[Publication Type] NOT ("animals"[MeSH Terms] NOT "humans"[MeSH Terms])

#3 #1 and #2

Supplementary Data S2. Literature Search Strategy for Cochrane CENTRAL

#1 (MeSH descriptor: [Dyslipidemias] explode all trees) OR (dyslipid*:ti,ab,kw OR "dyslipoproteinemia*":ti,ab,kw OR "hyperlipemia*":ti,ab,kw OR "hyperlipid*":ti,ab,kw OR "lipemia*":ti,ab,kw OR "lipidemia*":ti,ab,kw OR "cholesteremia":ti,ab,kw OR "cholesterinemia":ti,ab,kw OR "elevated cholesterol*":ti,ab,kw OR "high cholesterol level*":ti,ab,kw OR "hyper beta lipoproteinemia":ti,ab,kw OR "hypercholesteremia*":ti,ab,kw OR "hypercholesterol*":ti,ab,kw) OR (MeSH descriptor: [Diabetes Mellitus] explode all trees) OR (diabet*:ti,ab,kw) OR (MeSH descriptor: [Obesity] explode all trees) OR ("adiposi*":ti,ab,kw OR "corpulenc*":ti,ab,kw OR "fatness":ti,ab,kw OR "obesity":ti,ab,kw OR "overweight":ti,ab,kw)

#2 (MeSH descriptor: [Fatty Acids] explode all trees) OR ("aliphatic NEAR/0 acid":ti,ab,kw OR "aliphatic NEAR/0 acids":ti,ab,kw OR "fa":ti,ab,kw OR "fatty NEAR/1 acid":ti,ab,kw OR "fatty NEAR/1 acids":ti,ab,kw OR "scfa":ti,ab,kw OR "vfa":ti,ab,kw OR "acid short chain fatty":ti,ab,kw OR "animal fat*":ti,ab,kw OR "ba":ti,ab,kw OR "butanoic acid*":ti,ab,kw OR "butyric acid*":ti,ab,kw OR "acetic acid*":ti,ab,kw OR "ethanoic acid*":ti,ab,kw OR "vinegar":ti,ab,kw) OR (MeSH descriptor: [Triglycerides] explode all trees) OR (tg:ti,ab,kw OR "triacylglycerol*":ti,ab,kw OR "triglyceride*":ti,ab,kw)

#3 ((MeSH descriptor: [Nutrition Therapy] explode all trees) OR ("nutrition*":ti,ab,kw) OR (MeSH descriptor: [Diet Therapy] explode all trees) OR (diet*:ti,ab,kw OR "food*":ti,ab,kw)) OR ((MeSH descriptor: [Diet, Vegetarian] explode all trees) OR (plant based nutrition:ti,ab,kw OR "vegetarian*":ti,ab,kw OR "vegan*":ti,ab,kw OR "macrobiotic diet*":ti,ab,kw OR "diet macrobiotic":ti,ab,kw OR "fat control diet":ti,ab,kw) OR (MeSH

descriptor: [Dietary Fats] explode all trees) OR (dietary NEAR/0 fat:ti,ab,kw OR "dietary NEAR/0 fats":ti,ab,kw OR "dietary oil*":ti,ab,kw))

#4 (MeSH descriptor: [Mortality] explode all trees) OR (MeSH descriptor: [Cause of Death] explode all trees) OR (mortalit*:ti,ab,kw OR "death":ti,ab,kw OR "fatalit*":ti,ab,kw OR "lethalit*":ti,ab,kw) OR (MeSH descriptor: [Stroke] explode all trees) OR (cerebral crisis:ti,ab,kw OR "cerebral vascular accident*":ti,ab,kw OR "cerebrovascular accident*":ti,ab,kw OR "brain vascular accident*":ti,ab,kw OR "vascular accident brain":ti,ab,kw OR "stroke*":ti,ab,kw OR "apoplectic fit":ti,ab,kw OR "apoplectic ictus":ti,ab,kw OR "apoplectic seizure":ti,ab,kw OR "apoplectic cerebral":ti,ab,kw OR "apoplectic attack*":ti,ab,kw OR "apoplexy":ti,ab,kw OR "cva":ti,ab,kw OR "cvas":ti,ab,kw OR "ais":ti,ab,kw OR "anterior cerebral circulation infarction":ti,ab,kw OR "brain infarct*":ti,ab,kw OR "infarct brain":ti,ab,kw OR "infarcts brain":ti,ab,kw OR "infarction brain":ti,ab,kw OR "infarcted brain":ti,ab,kw OR "brain venous infarction*":ti,ab,kw OR "ci":ti,ab,kw OR "cerebral infarct*":ti,ab,kw OR "watershed infarct*":ti,ab,kw) OR (MeSH descriptor: [Risk] explode all trees) OR (MeSH descriptor: [] explode all trees and with qualifier(s): [adverse effects - AE])

#5 (MeSH descriptor: [Cardiovascular Diseases] explode all trees) OR ("cardiovascular disease*":ti,ab,kw OR "cvd":ti,ab,kw OR "ami":ti,ab,kw OR "cardiac infarction":ti,ab,kw OR "cardiovascular stroke*":ti,ab,kw OR "coronary infarction*":ti,ab,kw OR "heart attack*":ti,ab,kw OR "mi":ti,ab,kw OR "myocardial infarct*":ti,ab,kw OR "omi":ti,ab,kw OR "ihd":ti,ab,kw OR "ischemic cardiomyopathy":ti,ab,kw OR "ischemic heart disease*":ti,ab,kw OR "myocardial ischemia*":ti,ab,kw OR "heart disease ischemic":ti,ab,kw OR "ischemia myocardial":ti,ab,kw OR "ap":ti,ab,kw OR "angina pectoris":ti,ab,kw OR

"angor pectoris":ti,ab,kw OR "stenocardia*":ti,ab,kw OR "cad":ti,ab,kw OR "chd":ti,ab,kw OR "coronary artery disease*":ti,ab,kw OR "coronary disease*":ti,ab,kw OR "coronary heart disease*":ti,ab,kw OR "coronary syndrome*":ti,ab,kw OR "acs":ti,ab,kw OR "hypertension":ti,ab,kw OR "high blood pressure*":ti,ab,kw OR "hypertonia*":ti,ab,kw OR "ht":ti,ab,kw OR "blood pressure high":ti,ab,kw OR "blood clot*":ti,ab,kw OR "prethrombotic state":ti,ab,kw OR "thrombose*":ti,ab,kw OR "thrombosis":ti,ab,kw OR "thrombus":ti,ab,kw OR "thromboembolism*":ti,ab,kw OR "embolism*":ti,ab,kw OR
"embolus":ti,ab,kw OR "emboli":ti,ab,kw)

#6 (MeSH descriptor: [Fatty Acids] explode all trees) OR (aliphatic NEAR/0 acid:ti,ab,kw OR "aliphatic NEAR/0 acids":ti,ab,kw OR "fa":ti,ab,kw OR "fatty NEAR/1 acid":ti,ab,kw OR "fatty NEAR/1 acids":ti,ab,kw OR "scfa":ti,ab,kw OR "vfa":ti,ab,kw OR "acid short chain fatty":ti,ab,kw OR "animal fat*":ti,ab,kw OR "ba":ti,ab,kw OR "butanoic acid*":ti,ab,kw OR "butyric acid*":ti,ab,kw OR "acetic acid*":ti,ab,kw OR "ethanoic acid*":ti,ab,kw OR "vinegar":ti,ab,kw) OR (MeSH descriptor: [Triglycerides] explode all trees) OR (tg:ti,ab,kw OR "triacylglycerol*":ti,ab,kw OR "triglyceride*":ti,ab,kw)

#7 (MeSH descriptor: [Nutrition Therapy] explode all trees) OR (nutrition*:ti,ab,kw) OR (MeSH descriptor: [Diet Therapy] explode all trees) OR ("diet*":ti,ab,kw OR "food*":ti,ab,kw)

#8 (MeSH descriptor: [Diet, Vegetarian] explode all trees) OR (plant based diet*:ti,ab,kw OR "plant based nutrition":ti,ab,kw OR "vegetarian*":ti,ab,kw OR "vegan*":ti,ab,kw OR "macrobiotic diet*":ti,ab,kw OR "diet macrobiotic":ti,ab,kw OR "fat control diet":ti,ab,kw)

OR (MeSH descriptor: [Dietary Fats] explode all trees) OR (dietary NEAR/0 fat:ti,ab,kw OR "dietary NEAR/0 fats":ti,ab,kw OR "dietary oil*":ti,ab,kw)

#9 (Randomized Controlled Trial:pt)

#10 (MeSH descriptor: [Animal] explode all trees) NOT (MeSH descriptor: [Human] explode all trees)

#11 #1 AND #2 AND #3 AND #4 OR #5 AND #6 AND #7 OR #8 AND #9 NOT #10

Supplementary Data S3. Literature Search Strategy for ICHUSI

After translation

#1 (Dyslipidemia/TH) or (Hypertension/TH) or (Diabetes/TH) or (Obesity/TH) or (Cardiovascular Disease/TH)

#2 (((Triglycerides/TH) or (Fatty Acid/TH) or (Saturated Fatty Acid/TA) or (Animal Fat/TA)) and ((Nutrition/TH) or (Diet Therapy/TH))) or ((Vegetarian/TH) or (Dietary Fat/TH) or (Low-fat diet/TA))

#3 (RD=Randomized Controlled Trial)

#4 #1 and #2 and #3
